# Epigenome‐wide association study of prostate cancer in African American men identified differentially methylated genes

**DOI:** 10.1002/cam4.70044

**Published:** 2024-08-20

**Authors:** Anders Berglund, Kosj Yamoah, Steven A. Eschrich, Rana Falahat, James J. Mulé, Sungjune Kim, Jaime Matta, Julie Dutil, Gilberto Ruiz‐Deya, Carmen Ortiz Sanchez, Liang Wang, Hyun Park, Hirendra N. Banerjee, Tamara Lotan, Kathryn Hughes Barry, Ryan M. Putney, Seung Joon Kim, Clement Gwede, Jacob K. Kresovich, Youngchul Kim, Hui‐Yi Lin, Jasreman Dhillon, Ratna Chakrabarti, Jong Y. Park

**Affiliations:** ^1^ Department of Biostatistics and Bioinformatics H. Lee Moffitt Cancer Center Tampa Florida USA; ^2^ Department of Radiation Oncology H. Lee Moffitt Cancer Center Tampa Florida USA; ^3^ Department of Immunology H. Lee Moffitt Cancer Center Tampa Florida USA; ^4^ Department of Radiation Oncology Mayo Clinic Alix College of Medicine and Health Sciences Jacksonville Florida USA; ^5^ Department of Basic Sciences Ponce Research Institute, Ponce Health Sciences University‐School of Medicine Ponce Puerto Rico; ^6^ Department of Tumor Biology H. Lee Moffitt Cancer Center Tampa Florida USA; ^7^ Department of Cancer Epidemiology H. Lee Moffitt Cancer Center Tampa Florida USA; ^8^ Natural, Pharmacy and Health Sciences Elizabeth City State University Elizabeth City North Carolina USA; ^9^ Johns Hopkins University Baltimore Maryland USA; ^10^ Department of Epidemiology and Public Health University of Maryland School of Medicine Baltimore Maryland USA; ^11^ Division of Pulmonology, Department of Internal Medicine, Seoul St. Mary's Hospital, College of Medicine The Catholic University of Korea Seoul Republic of Korea; ^12^ Department of Health Outcome and Behavior H. Lee Moffitt Cancer Center Tampa Florida USA; ^13^ Biostatistics and Data Science Program, School of Public Health Louisiana State University School of Medicine New Orleans Louisiana USA; ^14^ Department of Pathology H. Lee Moffitt Cancer Center Tampa Florida USA; ^15^ Burnett School of Biomedical Sciences University of Central Florida Orlando Florida USA; ^16^ Program in Oncology University of Maryland Greenebaum Comprehensive Cancer Center Baltimore Maryland USA

**Keywords:** epidemiology, epigenetics, methylation, prostate cancer

## Abstract

**Introduction:**

Men with African ancestry have the highest incidence and mortality rates of prostate cancer (PCa) worldwide.

**Methods:**

This study aimed to identify differentially methylated genes between tumor vs. adjacent normal and aggressive vs. indolent PCa in 121 African American patients. Epigenome‐wide DNA methylation patterns in tumor DNA were assessed using the human Illumina Methylation EPIC V1 array.

**Results:**

Around 5,139 differentially methylated CpG‐sites (q < 0.01, lΔβl > 0.2) were identified when comparing normal vs. tumor, with an overall trend of hypermethylation in prostate tumors.  Multiple representative differentially methylated regions (DMRs), including immune‐related genes, such as *CD40*, *Galectin3*, *OX40L*, and *STING*, were detected in prostate tumors when compared to adjacent normal tissues. Based on an epigenetic clock model, we observed that tumors’ total number of stem cell divisions and the stem cell division rate were significantly higher than adjacent normal tissues. Regarding PCa aggressiveness, 2,061 differentially methylated CpG‐sites (q < 0.05, lΔβl > .05) were identified when the grade group (GG)1 was compared with GG4/5. Among these 2,061 CpG sites, 155 probes were consistently significant in more than one comparison. Among these genes, several immune system genes, such as *COL18A1*, *S100A2*, *ITGA4*, *HLA‐C*, and *ADCYAP1*, have previously been linked to tumor progression in PCa.

**Conclusion:**

Several differentially methylated genes involved in immune‐oncologic pathways associated with disease risk or aggressiveness were identified. In addition, 261 African American‐specific differentially methylated genes related to the risk of PCa were identified. These results can shedlight on potential mechanisms contributing to PCa disparities in the African American Population.

## 1 INTRODUCTION

The risk of prostate cancer (PCa) and PCa‐specific mortality rates are different in different racial groups. Environmental and biological factors, such as epigenetics, may contribute to these health disparities. DNA methylation levels may affect cancer risk and progression. In prostate tumor tissues, abnormal DNA methylation levels are frequent molecular changes. The biological impact of hypermethylation has been related to PCa‐specific death, metastasis, and recurrence by downregulation of tumor suppressor genes.^1^, ^2^, ^3^, ^4^, ^5^

Differential DNA methylation is associated with prostate carcinogenesis, and progression.^6^, ^7^ We and others reported differentially methylated genes between prostate tumors and normal biospecimens, as well as between aggressive and indolent cases using an epigenome‐wide association study approach with Illumina EPIC arrays.^8^, ^9^, ^10^, ^11^, ^12^ Investigation for racial‐specific methylation profiles is warranted to find potential mechanisms for health disparity.^13^ Previous studies reported that the gene expression of prostate tumor tissues showed significant differences in tumor immunobiology in African American men (AAM) compared to European American men (EAM).^14^ Allelic variants found in AAM can enhance gene expression, often leading to an immunosuppressive tumor microenvironment. These allelic variants can potentially contribute to disease aggressiveness and poor outcomes in AAM when compared with EAM.^15^

Age is one of the main risk factors for PCa. The mitotic age and the total number of cell divisions are correlated with cancer development.^16^ The aging tissues show stochastic DNA methylation drift, thus imperfect maintenance of epigenetic levels.^17^ These drifts induce aging stem cell exhaustion and focal proliferative defects, possibly leading to carcinogenesis.^18^, ^19^ DNA methylation biomarkers for aging, or epigenetic clocks, are based on DNA methylation data to predict chronological age or mortality risk.^20^, ^21^, ^22^, ^23^ DNA methylation age generated from these clocks is correlated with disease and all‐cause mortality^20^, ^24^ and cancer survival.^25^, ^26^ We used the HypoClock method to compare the stem cell division rate and the total number of stem cell divisions between the tumor and adjacent tissues.^26^

The goal of this study was to identify differentially methylated genes in tumor tissues and aggressive PCa cases in AAM. Differential methylation in immune‐oncologic genes in PCa of AAM and EAM was also explored. In addition, several of the differentially methylated genes identified are involved in immune pathways, and several of these genes have previously been investigated for their roles in PCa risk or tumor aggressiveness. In summary, an epigenome‐wide association study was performed using paired tumor and normal tissue from 121 African American (AA) men with PCa. In this study, methylated immune genes, which can be biomarkers for the aggressive PCa in AAM were identified. Our results may provide information on potential mechanisms responsible for health disparities in the AA population studied.

## 2 MATERIALS AND METHODS

### 2.1 Study population, clinical data, and tissue sample collection

The Institutional Review Board of the State of Florida DOH (#160030MOFF) approved this study. All study participants self‐identified as African American, or Black, were diagnosed with histologically confirmed PCa between January 2013 and December 2017, resided within Florida at the time of diagnosis, and provided written informed consent. Eligible individuals were identified, and patients' clinical and epidemiological data were collected in collaboration with the Florida Cancer Data System, which is the Florida State Cancer Registry supported by the Florida DOH. Formalin‐fixed paraffin‐embedded (FFPE) prostate tumor and adjacent paired tissue samples were obtained from the hospitals where the patients were treated. Tumors from principal component analysis (PCA) patients were classified as aggressive (GG4/5), intermediate (GG2/3), or indolent cases (GG1) based on Grade Groups.

### 2.2 DNA methylation analysis

#### 2.2.1 Epigenome‐wide profiling using Illumina EPIC methylation array

The Illumina Methylation EPIC V1 BeadChip was used for methylation levels using genomic DNA samples from FFPE tissues, as previously described.^12^ These epigenetic assays were performed at the Molecular Genomics Core at Moffitt Cancer Center (Tampa, FL). Genomic DNA was extracted from the tumor area after the pathologist's evaluation. The quality of genomic DNA was evaluated by DNA integrity numbers (DINs). The average DIN was 4.75 ± 0.79.

#### 2.2.2 Bioinformatic analysis of data obtained from Illumina EPIC methylation assay

The minfi (version 1.44.0) Bioconductor package for R (version 4.2.1) was used to read raw fluorescence intensity data (IDAT) files.^27^, ^28^ Minfi's implementation for estimating *p*‐values was used. Normalization was performed using the *minfi* preprocessNoob function, which performs the NOOB method^29^ for background correction, as well as a dye‐bias normalization. *β*‐values were estimated using the intensity of the methylated signal divided by the sum of both methylated and unmethylated signals, *β* = Mech/(Meth + Unmeth). Underperforming CpG probes were deleted based on the recommendation by Zhou et al.^30^ To identify outliers, and to visualize data quality and potential batch effects, Histograms of *β*‐values, the number of missing values, and PCA were performed. Values of *β* with a corresponding detection *p* > 0.01 were considered as missing values.

#### 2.2.3 CSG PCA and ICG PC2 calculations

The PCA model, based on all TCGA tumor types and the immune synapse genes derived in Berglund et al.,^31^ was applied to our data. The first principal component, PC1, is linked to co‐stimulatory genes (CSGs), while the second principal component is linked to immune checkpoint gene (ICG).

#### 2.2.4 Visualization of genes

To visualize the methylation level for all CpG‐probes within a gene, we used gene structure methylation (GSM) plots, as previously described,^32^ were used. In short, the GSM plot shows the methylation level along the *x*‐axis using boxplots, while the *y*‐axis shows the CpG‐probe location and CpG‐probe ID. The location of CpG‐islands is indicated by the first vertical bar, while the second vertical bar shows the gene structure.

#### 2.2.5 Immune cell deconvolution

We estimated the immune cell composition using the extended flow‐sorted method by Salas et al.^33^ The FlowSorted.BloodExtended.EPIC package contains methylation data generated on the Illumina HumanMethylationEPIC array for 12 different cellular populations, such as natural killer lymphocytes (NK), T regulatory cells (Treg), B lymphocytes memory (Bmem), neutrophils (Neu), monocytes (Mono), eosinophils (Eos), T helper lymphocytes memory (CD4mem), basophils (Bas), B lymphocytes naïve (Bnv), T helper lymphocytes naïve (CD4nv), T cytotoxic lymphocytes naïve (CD8nv), T cytotoxic lymphocytes memory (CD8mem), and. These cellular reference samples were first normalized with our methylation data using the *minfi* preprocessNoob function. Then, 1200 CpG probes that were IDOL‐optimized^34^ based on their differing methylation signatures across 12 cell types were used for the deconvolution into cellular proportions, according to Houseman et al.^35^ These IDOL‐optimized probes were provided with the FlowSorted.BloodExtended.EPIC package as the IDOLOptimizedCpGsBloodExtended object.

#### 2.2.6 PCA model using all CpG‐probes

The PCA model was derived using all CpG‐probes, excluding CpG‐probes with >20% missing values and no scaling of the individual CpG‐probes.

#### 2.2.7 Differentially methylated CpG‐probes

A two‐sided Student *t*‐test with unequal variance was used for a group comparison. The derived *p*‐value was adjusted for multiple testing to derive *q*‐values using Storey's method.^36^ To be considered significant, the average difference (Δ*β*‐value) between the two groups had to be greater than 0.2 for tumor versus normal and 0.1 for aggressive versus indolent cases.

#### 2.2.8 Degree of hypermethylation

(Σ Hyper − Σ Hypo)/Σ Total. The degree of hypermethylation was calculated using the normalized Euclidian distance for the Δ*β*‐value and −log_10_(*q*‐value) for each probe. The calculated value ranges between −1 and 1.

#### 2.2.9 TCGA prostate (PRAD) data

The TCGA prostate dataset was downloaded as raw IDAT's and normalized using the method mentioned above. Samples of European ancestry were selected based on the publication by Carrot‐Zhang et al.^37^ using the consensus ancestry.

#### 2.2.10 Estimation of epigenetic clocks

We used the epiTOC2 “HypoClock” method to calculate the stem cell division rate and the total number of stem cell divisions between the tumor and adjacent tissues.^26^ The Sankey diagram was generated using SankeyMATIC (sankeymatic.com). All statistical calculations and visualization were done with MATLAB R2022b (The MathWorks Inc. Natick, MA).

## 3 RESULTS

### 3.1 Epidemiological and clinical information of characteristics of participants

The average age at diagnosis for the AAM with PCa included in this study was 58.6 years. Almost 19% (*n* = 23) of PCa patients are a GG4/5 and were classified as an aggressive disease. Twenty‐one percent of patients (*n* = 26) had a GG1 which were considered indolent. A total of 72 patients had a GG2/3 and were classified as intermediate. A different distribution in grade and stage was observed among the three study groups (*p* < 0.0001). We did not observe statistically significant differences among the three groups in terms of mean age at diagnosis, mean body mass index (BMI), tobacco use, marital status, and mean prostate‐specific antigen (PSA) level (Table 1).

**TABLE 1:** Clinicopathological and demographic characteristics of African American men (*n* = 121) with prostate cancer that were studied.

	Total (*n* = 121)	Indolent (*n* = 26)	Intermediate (*n* = 72)	Aggressive (*n* = 23)	*p* value
Age at diagnosis	58.6 ± 7.6^a^	56.2 ± 7.1	59.4 ± 7.4	59.4 ± 8.2	0.159
PSA	8.8 ± 12.9	8.7 ± 16.3	7.7 ± 10.8	12.9 ± 13.0	0.244
Gleason score					<0.0001
6	26	26	0	0	
7	72	0	72	0	
8	16	0	0	16	
9	7	0	0	7	
Grade					<0.0001
1	16	16	0	0	
2	77	10	65	2	
3	29	0	7	21	
TNM stage					<0.0001
1	67	24	36	6	
2	51	2	36	13	
3	3	0	0	3	
4	1	0	0	1	
Tobacco use					0.457
Never	73	15	41	16	
Current	14	2	10	2	
Former	23	8	11	4	
Unknown	12	1	10	1	
Marital status					
Single	25	5	15	5	0.962
Married	81	18	46	16	
Separated, divorced, widowed	15	3	10	2	
Unknown	1	0	1	0	
BMI	29.5 ± 6.2	30.1 ± 6.1	28.8 ± 6.2	31.0 ± 6.4	0.335

^a^ Values expressed as mean ± 1 S.D.

### 3.2 Differential methylation in genes between prostate tumor and adjacent normal tissues

**FIGURE 1:**
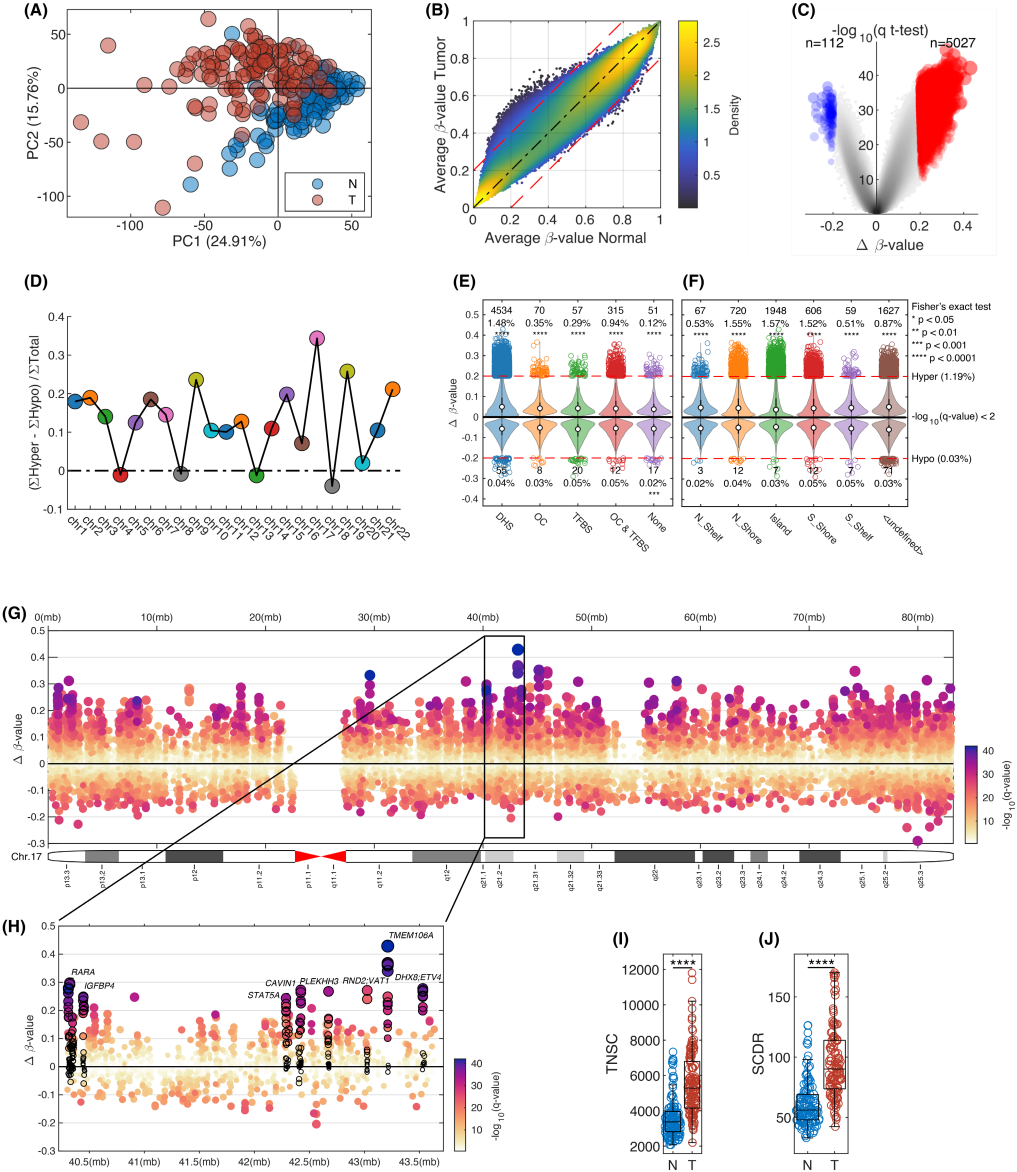
Hypermethylation in AA PCa compared to adjacent normal tissue. (A) A principal component analysis (PCA) using all CpG sites shows the separation of tumor (red circles) and normal tissue (blue circles). (B) Density scatter plot average *β*‐value for normal samples versus tumor samples. (C) Volcano plot comparing Normal versus tumor with Δ*β*‐value on the *x*‐axis and multiple tested corrected *p*‐value on the *y*‐axis. (D) Degree of hypermethylation across 22 chromosomes. (E) TFBS, Distribution of significant DNase hypersensitivity (DHS) CpG‐probes, and open chromatin (OC) probes, (F) Distribution of significant CpG‐probes across the different part of CpG‐islands. (G) Chromosome plot of methylation changes between normal and tumor for chromosome 17. Δ*β*‐value on the *y*‐axis, color based on *q*‐value, and size based Δ*β*‐value. (H) Zoomed in region of chromosome 17 (40,300,000–43,640,000) with a few selected genes highlighted (*ETV4*, *RARA*, *IGFBP4*, *TMEM106A*, *STAT5A*, *RND2*, *CAVIN1*, *PLEKHH3*, *VAT1*, and *DHX8*). Box plots comparing (I) TNSC per stem cell and (J) SCDR for normal versus tumor. ****p < 0.0001.

For quality control criteria, we used PCA, missing values, quality control analysis, and *β*‐value distributions. Four samples were excluded from the analysis since they did not meet these criteria, resulting in 238 samples. The PCA separated the tumor and adjacent tissues (Figure 1A), indicating a different methylation level in tumors than adjacent tissues. We presented the average value for the tumor and adjacent samples using a scatter density graph in Figure 1B. This figure suggested that the average value for tumor tissues is higher than that of adjacent normal tissues.

After comparison between adjacent normal and tumor tissues, a volcano plot was generated to visualize the different methylation between the two groups showing average change (Δ*β*‐value) and statistical significance (*q*‐value). In addition, using the FDR (*q* < 0.01) and fold change (Δ*β*‐value >0.2), we identified 5139 differentially methylated CpG sites; most CpG sites (98%) were hypermethylated (*n* = 5027) in tumor samples, based on this analysis (Figure 1C).

The degree of increased hypermethylation in tumor samples was compared to normal samples across the 22 human chromosomes (Figure 1D). While most chromosomes showed hypermethylation in tumors, chromosome 17 had the highest degree of hypermethylation. Different functional locations, that is, transcription factor binding sites (TFBS), DNase hypersensitivity CpG (DHS), and open chromatin (OC), were used to compare fold change value (Δ*β*‐value) for hypermethylated CpG sites (Figure 1E). Significantly methylated CpG sites were detected in all locations, especially DHS. The differentially methylated CpG site locations were also explored (Figure 1F). As expected, the gene regions most differentially methylated on the CpG islands were the promoter regions. Figure 1G,H showed the Δ*β*‐value between normal and tumor across all CpG‐probes on this chromosome 17. The genes affected mainly by hypermethylation in this chromosome 17 were at a region that encompasses various genes, such as *RARA*, *PLEKHH3*, *IGFBP4*, *TMEM106A*, *STAT5A*, *CAVIN1*, *RND2*, *VAT1*, *DHX8*, and ETV*4* (Figure 1G,H).

### 3.3 Epigenetic clocks in tumor and adjacent tissues

High mitotic age increases the risk of mutations,^19^ which correlates with the risk of cancer development.^16^ We estimated the methylated cell fraction in 163 CpG probes using a mathematical expression based on Teschendorff's epigenetic mitotic clocks method.^26^ We determined that TNSC based on age at diagnosis and the prostate‐specific probability of new and baseline methylation. Our results showed higher SCDR and TNSC in tumor tissues (*p* < 0.0001) (Figure 1I,J).

### 3.4 Differential methylation in immune genes between prostate cancer tumor and adjacent normal tissues

**FIGURE 2:**
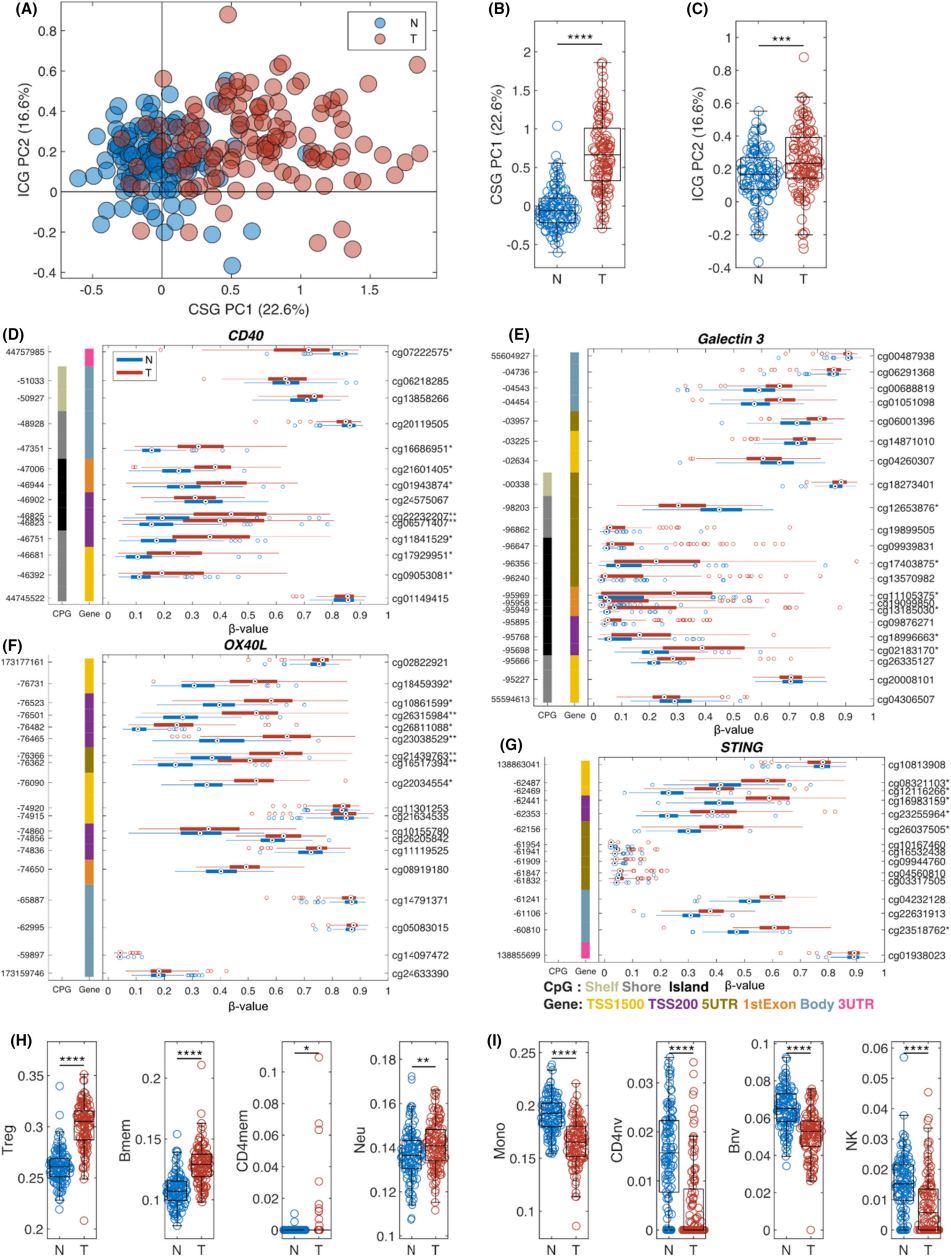
Epigenetic changes in immune genes for AA PCa. (A) Scatter plot of prediction using immune synapse principal component analysis (PCA) model with adjacent normal tissue in blue and prostate cancer (PCa) in red. Boxplots of normal and tumor samples for co‐stimulatory genes (CSGs) (B) and immune checkpoint gene (ICG) (C). Several genes show a significant difference in methylation for the immune synapse genes (***p < 0.001, ****p < 0.0001). Individual gene structure methylation (GSM)‐plots for (D) *CD40*, (E) *Galectin 3*, (F) *OX40L*, and (G) *STING* (*q < 0.05 and |Δ*β*| > 0.1, **q < 0.01 and |Δ*β*| > 0.2). Boxplots comparing normal versus tumor for immune cell types with an increased amount in (H), and decreased amount in (I) in tumor compared to normal (**p < 0.01, ***p < 0.001, ****p < 0.0001).

Tumor immune pathways are affected by immune‐synapse between effector T cells and antigen‐presenting cells. In addition, immune surveillance was evaded for carcinogenesis.^38^ The immune synapse PCA model was applied to 238 samples using the *β*‐values for methylation levels for all CpG sites for CSGs immune checkpoint genes (ICGs) (Figure 2A). Significantly different methylation levels were found between tumor and adjacent tissues in the immune synapse genes, thus CSGs (*p* < 0.0001) and ICGs (*p* < 0.001) (Figure 2B,C). GSM plots were presented for representative immune genes with significant changes in methylation patterns, including *CD40* (Figure 2D), *galectin 3* (Figure 2E), *OX40L* (Figure 2F), and *STING* (Figure 2G) (*q < 0.05 and |Δ*β*| > 0.1, **q < 0.01 and |Δ*β*| > 0.2). We observed that 57% (8/14) of CpG probes in *CD40* were hypermethylated in tumor tissues (Figure 2D).

Immune cell types were also compared between tumors and adjacent normal tissues. Memory CD4^+^ T cells (CD4mem), regulatory T cells (Treg), memory B cells (Bmem), and neutrophils are significantly higher in tumors (Figure 2H). NK cells. Monocytes (Mono), naïve CD4^+^ naïve B cells (Bnv), and T cells (CD4nv) are significantly reduced in tumors compared to adjacent tissues (Figure 2I).

### 3.5 Differentially methylated genes identified in only African American patients

**FIGURE 3:**
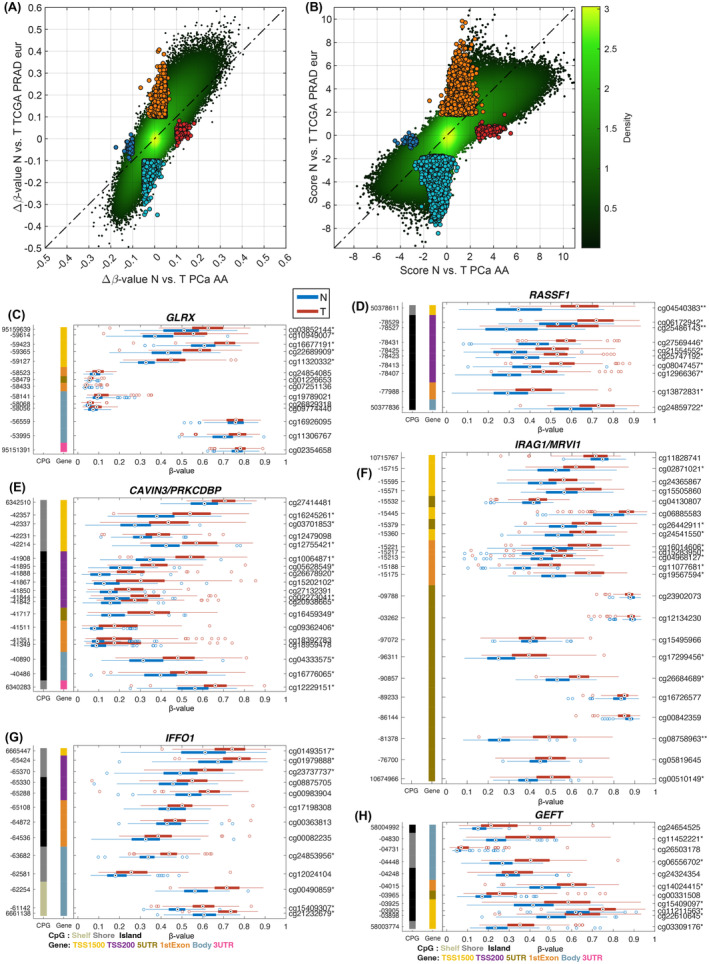
Comparing methylation changes in PCa AA to European cohorts. Density scatter plot comparing Δ*β*‐value (A) and score (B) (combining Δ*β*‐value and −log_10_(*p*‐value)) for TCGA PRAD European (*y*‐axis) and our PCa AA (*x*‐axis) between normal and tumor. Highlighted markers indicate unique CpG‐probes that are only significant in one of the groups. Individual GSP plots for genes that are only significant in our PCa AA cohort when comparing normal versus tumor, (C) *GLRX*, (D) *RASSF1*, (E) *CAVIN3*, (F) *IRAG1*, (G) *IFFO1*, and (H) *GEFT* (*q < 0.05 and |Δ*β*| > 0.1, **q < 0.01 and |Δ*β*| > 0.2).

Additional comparisons were performed between the results obtained with our cohort of AAM with an EAM cohort using TCGA data. The results based on Δ*β*‐value and score (combining Δ*β*‐value and −log_10_(*p*‐value)) demonstrated an overall similarity in methylation changes between AAM and EAM cohorts (Figure 3A,B). However, 261 differentially methylated genes were identified only in tumors from AA patients. Among these genes, several AA‐specific methylated genes such as *GLRX*,^39^ *RASSF1*,^40^ *CAVIN3*,^41^ *IRAG1*,^42^ *IFFO1*,^43^ and *GEFT* ^44^ (Figure 3C–H) have been investigated for their potential roles in human cancers including PCa.

### 3.6 Association between differential methylation and aggressive PCa

**FIGURE 4:**
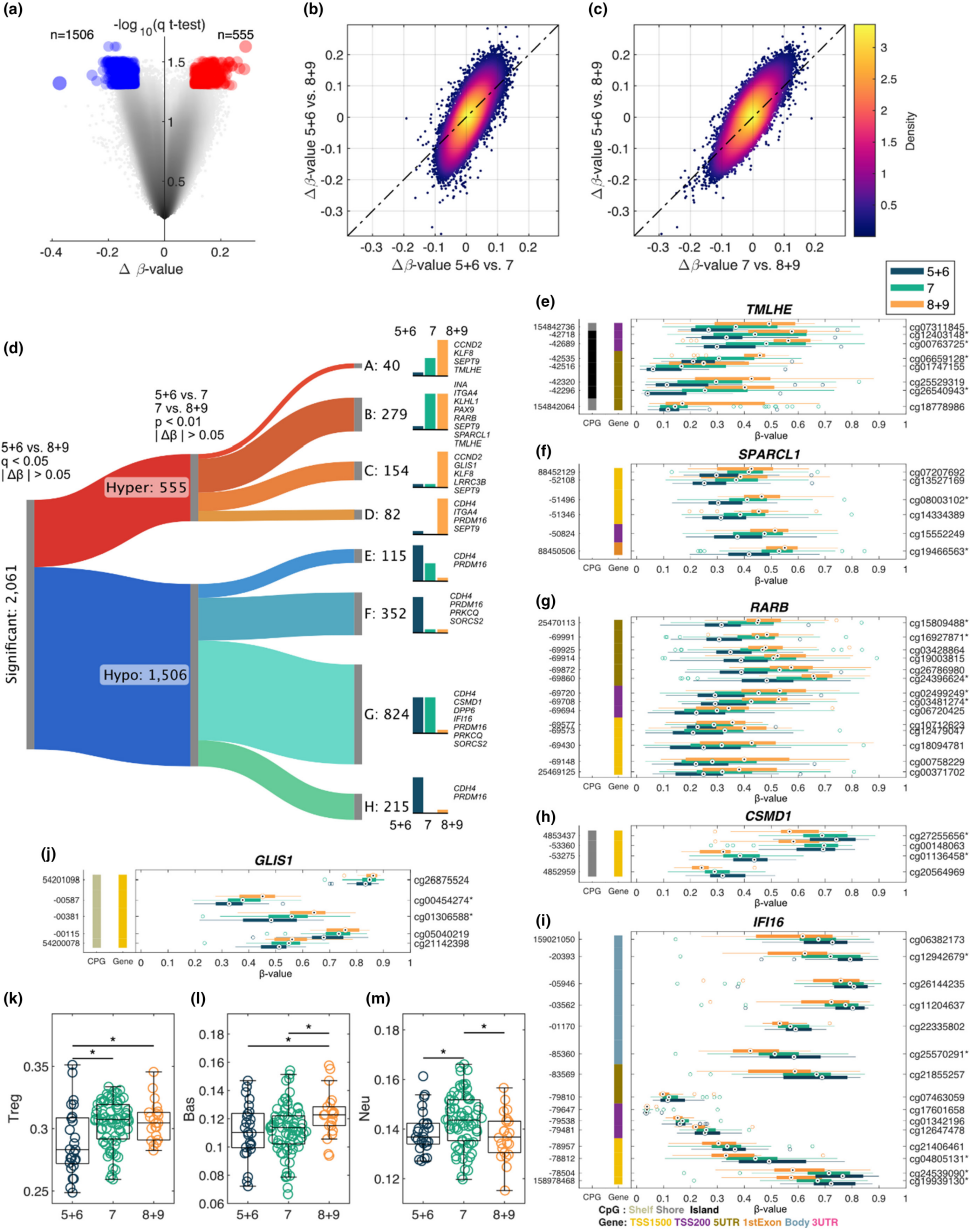
Methylation changes based on Gleason score. (a) Volcano plot describing the methylation changes between samples with GG1 versus GG4/5, *∆β*‐value on the *x*‐axis and multiple tested corrected *p*‐value on the *y*‐axis. Density scatter plot comparing the *∆β*‐value for GG1 versus GG4/5 and (b) *∆β*‐value for GG1 versus GG2/3 and (c) *∆β*‐value for GG2/3 versus GG4/5. (d) Sankey diagram classifying significant (GG1 vs. GG4/5) CpG‐probes into eight different categories based on their changes when comparing GG1 versus GG2/3 and GG2/3 versus GG4/5. A, hypermethylated CpG‐probes that are significant in both GG1 versus GG2/3 and GG2/3 versus GG4/5. B, hypermethylated CpG‐probes that are significant in GG1 versus GG2/3. C, hypermethylated CpG‐probes that are significant in GG2/3 versus GG4/5. D, hypomethylated CpG‐probes that are not significant in any of the other comparisons. E, hypo‐methylated CpG‐probes that are significant in both GG1 versus GG2/3 and GG2/3 versus GG4/5. F, hypermethylated CpG‐probes that are significant in GG1 versus GG2/3. G, hypomethylated CpG‐probes that are significant in GG2/3 versus GG4/5. H, hypomethylated CpG‐probes that are not significant in any of the other comparisons. Selected genes are listed for each category. gene structure methylation (GSM) plots comparing the methylation levels between the three groups for (e) *TMLHE*, (f) *SPARCL1*, (g) *RARB*, (h) *CSMD1*, (i) *IFI16*, (j) *GLIS1* (*q < 0.05 and |*∆β*| > 0.1 for GG1 vs. GG4 + 5). Boxplots comparing GG1 versus GG2/3 versus GG4/5 for immune cell types with (k) Treg, (l) Basophiles, and (m) Neutrophiles, *p < 0.05.

The association between differential methylation and PCa aggressiveness based on Gleason scores was also investigated. A volcano plot was generated by comparison between the two extreme groups thus, GG1 versus GG4/5 are presented in Figure 4a. We identified 2061 differentially methylated CpG sites (hypomethylated: 1506 and hypermethylated: 555), using *q*‐value (*q* < 0.05) and the mean difference (Δ*β*‐value >0.1) (Figure 4a). Density scatter plots based on Δ*β*‐value between GG1 versus GG2/3 and between G2/3 versus GG4/5 are presented in Figure 4b,c, respectively.

Using a Sankey graph, the two groups on the extremes (GG1 vs. GG4/5) were compared, and 2061 significant CpG‐probes were classified into eight categories. These categories describe both hyper‐ and hypomethylation and the different grade group areas (GG1 vs. GG2/3 and GG2/3 vs. GG4/5). Significantly hypermethylated CpG‐probes (*n* = 40) were identified in both GG1 versus GG2/3 and GG2/3 versus GG4/5 comparisons (Figure 4d, A), while 279 hypermethylated CpG probes were found in GG1 versus GG2/3 comparison (Figure 4d, B). Hypermethylated CpG‐probes (*n* = 154) were significant in GG2/3 versus GG4/5 (Figure 4d, C), while 82 hypermethylated CpG‐probes were not significant in GG1 versus GG2/3 but were significant in GG1and GG2/3 versus GG4/5 (Figure 4d, D). Significant hypomethylated CpG‐probes (*n* = 115) were found in both GG1 versus GG2/3 and GG2/3 versus GG4/5 (Figure 4d, E) while 352 hypomethylated CpG‐probes were noted to be significant in GG1 versus GG2/3 and GG4/5 (Figure 4d, F). Hypomethylated CpG‐probes (*n* = 824) were significant in GG1 and GG2/3 versus GG4/5 (Figure 4d, G), while 215 hypomethylated CpG‐probes were not significant in GG2/3 versus GG4/5 comparisons (Figure 4d, H). GSM plots were generated comparing methylation levels among three groups for *TMHLE* (Figure 4e), *SPARCL1* (Figure 4f). *RARB* (Figure 4g) and *GLIS1* (Figure 4j) showed hypermethylation in GG4/5 tumors. In contrast, *CSMD1* (Figure 4h) and *IFI16* (Figure 4I) showed hypomethylation in GG4/5 tumors (*q < 0.05 and |Δ*β*| > 0.1 for GG1 vs. GG4/5).

### 3.7 Differential distribution of immune cells associated with aggressive PCa

The distribution of immune cells, including Treg (Figure 4k), Basophiles (Figure 4l), and Neutrophiles (Figure 4m) was compared among the three risk groups (GG1, GG2/3, and GG4/5).

## 4 DISCUSSION

PCa‐specific mortality rate in AAM is 2.2 fold higher than EAM.^45^ However, the associations between racial disparity and epigenetic risk factors have not been fully investigated yet.^11^ In this study, a handful of differentially methylated genes, including immune‐related genes, were identified in aggressive tumors from AA men with PCa. Among 22 human chromosomes (Figure 1d), we found that chromosome 17 had the highest degree of hypermethylation as compared with normal prostate tissues. Some hypermethylated genes in chromosome 17 were *RARA*, *PLEKHH3*, *IGFBP4*, *TMEM106A*, *STAT5A*, *CAVIN1*, *RND2*, *VAT1*, *DHX8*, and *ETV4* (Figure 1g,h). Previous studies also support hypermethylation in chromosome 17 in PCa.^46^, ^47^

We identified 5139 CpG sites with a significant differential methylation pattern in the prostate tumor tissue, with 98% hypermethylated (Figure 1). We evaluated the differentially methylated genes' potential roles in immune oncologic pathways in PCa disparity in AAM. Our results suggest that AAM with PCa has AA‐specific differentially methylated immune‐related genes. We identified potential methylation biomarkers in the immune–oncological pathway, including *CD40*, *galectin 3*, *OX40L*, and stimulator of interferon genes (*STING*) (Figure 2). These genes were previously investigated for their roles in the risk and progression of PCa. The immunological response triggered by the cyclic GMP‐AMP synthase (cGAS)–STING pathway recently increased scientific interest. The carcinogenesis and its progression can be induced by dysregulation of the cGAS–STING pathway, which may influence antitumor immune reaction.^48^, ^49^ STING, a tumor suppressor gene, was proposed as a promising biomarker of PCa because STING is downregulated in prostate tumor tissues. Hypermethylation of *STING* may lead to low expression of STING, promoting cancer development.^50^ Differential DNA methylation profiles in PCa tumors from the AA population were reported previously.^51^

Stem cell division and epigenetic modifications, such as DNA methylation status, continue to change during PCa progression at different stages. Therefore, the DNA methylation‐based epigenetic clock provides essential information on the status of cancer progression. This epigenetic clock showed a consistent universal acceleration pattern in various tumor tissues.^52^ Based on DNA methylation analysis, the TNSC and SCDR were significantly higher in the tumor than those adjacent normal prostate tissues (Figure 1I,J).

DNA methylation levels were compared between tumor and adjacent tissues from our AA and TCGA EAM patients. Among 261 AA‐specific methylated genes, several genes were investigated for their potential roles in various human cancers, such as *CAVIN3* (Figure 3e). Methylation of the *CAVIN3* was reported in lung and breast cancers where CAVIN3 was suggested as a tumor suppressor protein.^53^, ^54^ Low expression of *CAVIN3* was related to hypermethylation of *CAVIN3* in breast tumors.^54^ In addition, the methylation level was correlated with disease progression and metastasis.^55^

We identified 2061 differentially methylated CpG probes after a comparison of AA patients with aggressive (GG4/5) versus indolent (GG1) PCa (Figure 4a). Significant CpG sites were classified into eight different classes based on methylation levers (hyper‐ or hypomethylation) and the different grade group areas (GG1 vs. GG2/3 and GG2/3 vs. GG4/5) using a Sankey graph. We identified several key genes in each category. Among these genes, *TMLHE* is involved in the epigenetic process in ovarian cancer (Figure 4e).^56^ *RARB* (Figure 4g), which showed hypermethylation in tumors with a higher Gleason score, was previously investigated in PCa. Woodson et al. reported that *RARB* was hypermethylated in prostate tumor samples.^57^ *RARB* hypermethylation was associated with an increased risk in AA men with PCa.^58^, ^59^ *SPARCL1* was identified as one of the critical diagnostic biomarkers for PCa (Figure 4f).^60^ *IFI16*, showed hypomethylation in higher Gleason score tumors (Figure 4i). The biological function of *IFI16* is to regulate cell growth arrest, which is associated with cellular senescence‐associated.^61^ Xin et al. reported that overexpression of *IFI16* induced the inhibition of cell growth in LNCaP and DU‐145 PCa cell lines.^62^ *IFI16* is one of the hypomethylated genes in early PCa.^63^ This *IFI16* is an immune‐related gene and induce antitumor activity. In animal study, *Ifi16* murine gene block carcinogenesis through high antitumor immunity. Hypomethylation of this gene was detected in circulating tumor cells of blood specimens.^63^ *CSMD1* was suggested as a candidate for a suppressor of PCa (Figure 4h).^64^

Emerging studies have shown that prostate tumors from AAM have low DNA damage repair gene expression, higher immune content, dysregulated immune‐related genomic markers, and increased cytokine and interferon levels.^65^, ^66^ All these factors contribute to tumor growth, dynamic anti‐tumor immunity, evade from immune surveillance, and increased risk of PCa progression and metastasis.^65^, ^66^ Whether the observed differential methylation of immune pathway genes such as *STING* and interferon genes in AA contributes to the differential expression profile of these genes and the ensuing therapeutic implications requires additional studies. These results may provide a foray into the mechanisms of treatment response supported by data from three different randomized studies in both localized and metastatic PCa showing that AA may respond better to immune‐modulatory therapy such as radiotherapy or immunotherapy.^67^, ^68^, ^69^

We are aware of limitations and strengths. First, although we carefully extracted DNA using the macrodissection method, we may have potential field effects in adjacent tissues. This may partially explain the overlapping DNA methylation values by tumor/normal status in the PCA analysis. Second, the lack of a validation set is a limitation. Third, more aggressive PCa cases were required for broader generalizations. Our next plan is to validate our results in a larger validation set and application of the liquid biopsy using cfDNA from blood from PCa patients. A major strength of our study is the identification of methylation patterns, related to PCa aggressiveness in the AA population. This information may contribute importantly to clinical management and help to find molecular mechanisms for racial disparity.

In conclusion, 2061 differentially methylated CpG sites were identified in tumors from AA men with aggressive PCa. Our results suggested the DNA methylation landscape of PCa among AA men. We also identified genes that influence multiple biological pathways in cancers, including the immune process. Our results suggested potential mechanisms underlying aggressive tumor phenotypes and aid in the prognostic evaluation of AA patients. Identification of unique DNA methylation patterns at diagnosis may introduce molecular biomarkers for physicians to select appropriate treatment strategies, especially for AA men with an aggressive type of PCa. Finally, our study paves the way for providing molecular tools to enable physicians to reduce racial disparities.

## AUTHOR CONTRIBUTIONS

**Anders Berglund:** Conceptualization (equal); formal analysis (equal); funding acquisition (equal); visualization (equal); writing – original draft (equal); writing – review and editing (equal). **Kosj Yamoah:** Conceptualization (equal); investigation (equal); writing – review and editing (equal). **Steven A. Eschrich:** Formal analysis (equal); investigation (equal); writing – review and editing (equal). **Rana Falahat:** Formal analysis (equal); investigation (equal); methodology (equal); writing – review and editing (equal). **James J. Mulé:** Conceptualization (equal); investigation (equal); supervision (equal); writing – review and editing (equal). **Sungjune Kim:** Conceptualization (equal); investigation (equal); writing – review and editing (equal). **Jaime Matta:** Conceptualization (equal); investigation (equal); writing – review and editing (equal). **Julie Dutil:** Conceptualization (equal); investigation (equal); writing – review and editing (equal). **Gilberto Ruiz‐Deya:** Conceptualization (equal); investigation (equal); writing – review and editing (equal). **Carmen Ortiz Sanchez:** Conceptualization (equal); investigation (equal); writing – review and editing (equal). **Liang Wang:** Conceptualization (equal); investigation (equal); writing – review and editing (equal). **Hyun Park:** Data curation (equal); investigation (equal); writing – review and editing (equal). **Hirendra N. Banerjee:** Conceptualization (equal); investigation (equal); writing – review and editing (equal). **Tamara Lotan:** Conceptualization (equal); investigation (equal); writing – review and editing (equal). **Kathryn H. Barry:** Conceptualization (equal); funding acquisition (equal); investigation (equal); writing – review and editing (equal). **Ryan M. Putney:** Data curation (equal); formal analysis (equal); investigation (equal); writing – review and editing (equal). **Seung Joon Kim:** Conceptualization (equal); investigation (equal); writing – review and editing (equal). **Clement Gwede:** Conceptualization (equal); investigation (equal); writing – review and editing (equal). **Jacob K. Kresovich:** Conceptualization (equal); formal analysis (equal); investigation (equal); visualization (equal); writing – original draft (supporting); writing – review and editing (equal). **Youngchul Kim:** Formal analysis (equal); investigation (equal); writing – review and editing (equal). **Hui‐Yi Lin:** Conceptualization (equal); formal analysis (equal); writing – review and editing (equal). **Jasreman Dhillon:** Investigation (equal); methodology (equal); visualization (equal); writing – review and editing (equal). **Ratna Chakrabarti:** Conceptualization (equal); investigation (equal); writing – original draft (equal); writing – review and editing (equal). **Jong Y. Park:** Conceptualization (lead); data curation (equal); funding acquisition (lead); investigation (lead); project administration (lead); resources (lead); supervision (lead); writing – original draft (lead); writing – review and editing (equal).

## Data Availability

The methylation data generated as part of this study are available at the Gene Expression Omnibus (GEO) under accession GSE269244.
